# Computational and experimental study of AC measurements performed by a double-nanohole plasmonic nanopore sensor on 20 nm silica nanoparticles

**DOI:** 10.1016/j.sbsr.2024.100694

**Published:** 2024-09-21

**Authors:** Homayoun Asadzadeh, Scott Renkes, MinJun Kim, George Alexandrakis

**Affiliations:** a University of Texas at Arlington, Bioengineering Department, Arlington, TX 76010, USA; b Southern Methodist University, Department of Mechanical Engineering, Dallas, TX 75275, USA

**Keywords:** Solid-state nanopores, Double nanohole, Plasmonic, Optical trapping, Conductance, AC, Phase shift, Computational model, COMSOL

## Abstract

A novel method of AC sensing is presented that uses a double nanohole (DNH) nanoaperture atop a solid-state nanopore (ssNP) to trap analytes and measure their optical and electrical properties. In this method analytes are propelled by an external applied voltage towards the sensor until they are trapped at the DNH-ssNP interface via a self-induced back action (SIBA) plasmonic force. We have previously named this method SIBA Actuated Nanopore Electrophoresis (SANE) sensing and have shown its ability to perform concurrent optical and DC electrical measurements. Here, we extend this method to AC sensing of 20 nm SiO_2_ (silica) nanoparticles, using voltage modulation over a wide range of frequencies applied on top of a baseline DC bias. The sensor was constructed using two-beam GFIS Focused Ion Beam (FIB) lithography, incorporating Ne FIB to mill the DNH and He FIB to drill a central 30 nm ssNP. We utilized COMSOL Multiphysics simulations to explore the multi-frequency AC current conductance properties of the silica nanoparticles trapped at the SANE sensor. These simulations computed conductance changes and phase shifts induced by the presence of the nanoparticle over an AC frequency range of 20 Hz to 100 kHz. Experimental measurements confirmed the trends seen in the computational data. Additional computational studies were then performed to dissect the underlying mechanisms driving the observed AC measurements. Looking forward, we aim to adapt this technology for probing therapeutic nanoparticles non-invasively, offering a promising tool for enhancing quality control of nanoparticle-mediated drug and gene delivery systems.

## 1. Introduction

Solid state nanopore (ssNP) sensors have become pivotal in label-free single-molecule detection and analysis, enabling precise discrimination between different analytes in solution. The operational principle of ssNPs is based on applying a voltage bias to drive analytes through them, leading to a decrease in ionic current as the nanopore is transiently blocked. The temporal profiles of these transient current blockade signals can then be used as fingerprints for recognizing analyte identity, conformational changes, or interaction dynamics, among many possible applications [1–10]. Nevertheless, the rapid transit of analytes through ssNPs limits the time during which analyte-relevant data can be collected to intervals in the tens of μs [11–19]. The integration of plasmonic optical trapping and sensing with ssNP technology marks a paradigm shift in the realm of biomolecular analysis, as it enables the immobilization of single nanoparticles and the study of their electrical properties, potentially extending the time available to characterize single analytes to attain more accurate characterization [20].

Translocation control of single nanoparticles and biomolecules has been previously demonstrated by ssNP integration with field effect transistors (FETs) [21], the nanopore electro-osmotic trap (NEOtrap) approach [22], and optical trapping by metallic nanoapertures that induce strong plasmonic confinement of light at low laser powers (mW range) [23]. Plasmonic optical trapping is based on the self-induced back action (SIBA) effect that keeps dielectric nanoparticles in the vicinity of light intensity maxima [24]. When a nanoaperture experiences dielectric loading from a trapped nanoparticle, a portion of the light scattered by the nanoparticle couples into the far field and creates a step-increase in transmitted amplitude intensity that is proportional to particle size [25–31]. In seminal contributions by Gordon et al. [32–43], a double nanohole (DNH) nanoaperture milled in gold (Au) was proposed for attaining efficient SIBA-mediated optical trapping.

Merging plasmonic optical trapping and sensing with ssNP-mediated electrical nanosensing, has been reported in recent years by us [44–46] and others [47–49]. In previous work, we have nanofabricated by He–Ne focused ion beam lithography a DNH nanoaperture with an ssNP at its center, which facilitated the integration of optical trapping by SIBA and concurrent label-free optical sensing with electrical ssNP detection of nanoparticles and biomolecules [50]. The DNH enabled plasmonically enhanced optical trapping of analytes just above the entrance of the underlying ssNP for several seconds [45]. This bimodal optical-electrical sensor technology was named SIBA-actuated nanopore electrophoresis (SANE) sensing. Merging optical and electrical sensing creates the additional consideration of how the laser beam could affect ssNP ion transport. A focused laser beam is known to create heating that alters ion transport in silicon nitride nanopores, both alone [51] and ones centered around an Au nanoantenna [52]. Interestingly, for the DNH sensor geometry, it has been shown that the effect of heating is minimal due to effective heat dissipation by the Au layer [53]. Nevertheless, electric charge accumulation induced by plasmons at the DNH tips could alter local ion transport [50].

One particular challenge that cannot yet be resolved today is that there is the inability to probe directly the inside of a nanoparticle with current ssNP or plasmonic sensing technologies. It is envisioned that this type of knowledge could be helpful to researchers in nanomedicine wanting to know how completely their nanoparticles are loaded with a drug or genetic material, or if they are empty. This work proposes to exploit the time interval during which single nanoparticles are optically trapped to perform multiple AC electrical impedance measurements, as a means of probing directly the electrical conductivity of the interior of a nanoparticle. The premise of this approach is that DC currents should be largely confined on the outside of a dielectric nanoparticle, envisaged as a spherical capacitor, whereas AC currents should be able, at least in part, to travel through the interior of the nanoparticle as well.

Specifically, this study describes COMSOL Multiphysics simulations on how multi-frequency AC current conductance measurements performed with the SANE sensor can probe the interior of a 20 nm silica (SiO_2_) nanoparticle. The performance of multi-frequency conductance measurements on a single nanoparticle is enabled by optical trapping that immobilizes the nanoparticle for a few seconds inside the trap. As a result, conductance measurements could be made for frequencies as low as 20 Hz and up to our data acquisition hardware bandwidth limit of 100 kHz. Multi-frequency conductance cannot be quantified with conventional ssNPs because analytes travel too rapidly through the sensor to allow enough time for multi-frequency measurements to take place. The simulations performed, enable modeling of AC current phase delay and conductance changes in the absence versus presence of a SiO_2_ nanoparticle inside the optical trap of the SANE sensor. Simulated measurements were compared with SANE sensor measurements when empty and when 20 nm SiO_2_ nanoparticles were optically trapped, closely matching experimental conditions. Although the simulations and measurements performed in this work focused on probing the interior of uniform spherical particles by AC sensing, after demonstrating proof of concept here, the next step will focus on applying this method to test the presence, or absence, of therapeutic loads inside biocompatible nanoparticles.

## 2. Materials and methods

### 2.1. Simulation setup and sensor geometry

**Fig. 1.**
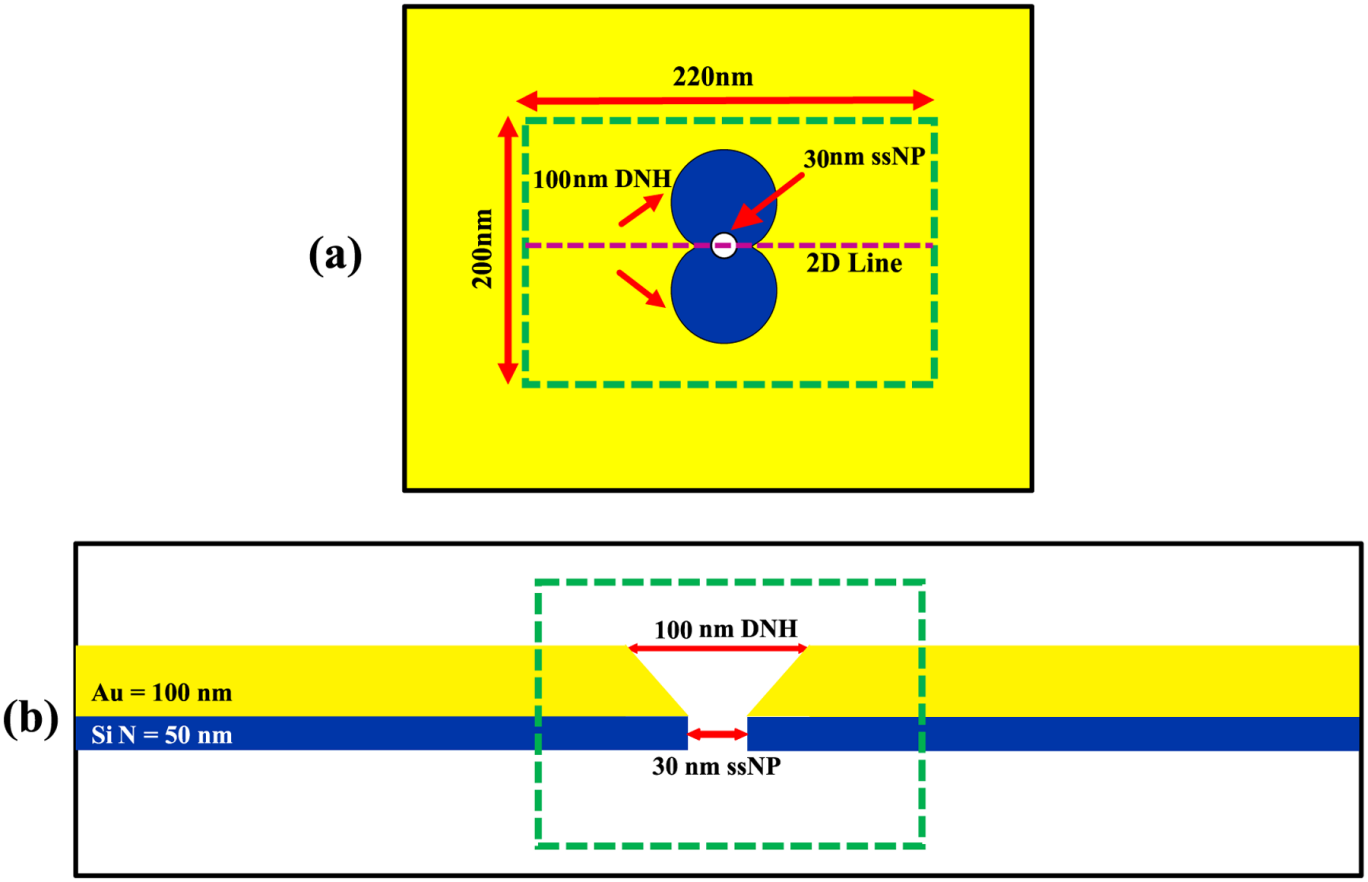
(a) Front side view and (b) cross-sectional view of the of SANE sensor geometry. The green-dashed rectangles indicate the boundaries of the COMSOL computational domain.

In COMSOL simulations the SANE sensor geometry was modeled as a DNH inside an Au layer (Fig. 1(a); 100 nm diameter circles, 100 nm Au layer thickness). A 30 nm diameter nanopore, located at the center of the DNH, penetrated through an underlying silicon nitride layer (SiN, 50 nm layer thickness) to allow analytes travel from *cis* to *trans*, as described previously [4]. Importantly, the physical sensor, was larger than the COMSOL-simulated sensor region (Fig. 1(a)–(b); green dashed lines) for computational economy. However, regions not included in the simulations were far away from the DNH and did not affect computational results. The nanofabrication steps of the physical implementation of the SANE sensor are described in Section 2.4, below.

Table 1 lists the SANE sensor layer thicknesses and the optical and electrical properties of the materials in each sensor layer assumed in the simulations. These values were selected to mimic as close as possible the corresponding physical sensor dimensions and properties. One approximation made was that the SiN layer in the physical sensor consisted of non-stoichiometric Si_x_N_y_, but for all simulations, the physical properties of the more well-characterized stoichiometric Si_3_ N_4_ were assumed instead. Layer thicknesses and optical and electrical properties for the sensor’s Au and Si_3_N_4_ layers, with Au data (including both real and imaginary parts of the refractive index, and the dielectric constant) sourced from Johnson and Christy [54]. For Si_3_N_4_, optical properties include only the real part of the refractive index from Philipp [55], with ***ε***_**1**_ approximated as n^2^ based on the absence of absorption data. The imaginary components were ignored in this case because optical absorption was negligibly small for the operational wavelength of 830 nm. To calculate the dielectric constant of Au at different frequencies, the Drude model [56] is typically employed, which considers the free electron response to electromagnetic fields. Additionally, the electrical conductivity of Au can also be computed by the Drude model, reflecting frequency-dependent changes due to electron mobility. For Si_3_N_4_, a non-metallic dielectric, the Lorentz model [57] is more suitable as it accounts for bound electron responses, resonant phenomena, and damping effects. Si_3_N_4_ exhibits very low electrical conductivity, which remains relatively constant across a wide range of frequencies.

Table 2 lists all relevant physical parameters assumed for the external laser illumination, electrode voltage bias and analyte concentrations, reflecting the same parameters used in the physical experiments for the empty sensor and 20 nm SiO_2_ nanoparticle trapping, as described previously [44].

### 2.2. Governing equations for the SANE sensor in the simulated region

The physics of liquids with ions dissolved in them can be approximated by three coupled classical equations: the Poisson equation relevant to electrostatics, the Nernst-Planck equation (NPE) describing ionic-flux, and the Navier-Stokes equation (NSE) governing the fluid flow [58]. The Poisson equation, which implements the electrostatic field in the Au and SiN layers of the sensor, relates the electric potential *V* to the charge distribution *ρ*_*v*_, and is given by:

(2–1) $$\nabla^{2}V=-\frac{\rho_{v}}{\epsilon_{0}\epsilon_{r}}$$

where *ϵ*_0_= 8.85e-12 F/m is the permittivity of free space and *ϵ*_*r*_ is the relative permittivity, inherent property of the material and *ρ*_*v*_ is volume charge density. The charge distribution (volume charge density) can relate the electrostatic field with the ionic concentration and space charge density which will be effective in forming the electric Debye layer on the nanopore’s wall in the SANE sensor. The related charge density equation can be represented in terms of ionic concentrations as:

(2–2) $$\rho_{v}=N_{A}e\sum z_{i}c_{i}$$

where *N*_*A*_= 6.0e23 *mol*^−1^ is the Avogadro’s constant, *e*= 1.6e-19C represents the elementary charge, *z*_*i*_ is the valence number and *c*_*i*_ is the molar concentration of species, *i*, in the electrolyte [59]. Monovalent electrolytes have been used in the majority of nanopore-based studies [60,61]. In the present study, 0.3 M Potassium Chloride (KCl) has been assumed as the electrolyte and pH was set at 7.4.

The space charge density of this salt, because of it is binary and monovalent nature, can be expressed as:

(2–3) $$\rho v=N_{A}e\left[C_{k+}-C_{Cl-}\right]$$

The flux *J*_*i*_ for each ionic species, *i*, is calculated using the Nernst-Planck equation:


(2–4) $$J_{i}=-D_{i}\nabla_{C\_i}-Z\left(D_{\_\text{iRT}}\right)FC_{i}\nabla V+uc_{i}$$

Looking at the equation closely, it may be deduced that the overall ionic flux is influenced by three components. The first part is caused by a concentration gradient, as described by Fick’s first law of diffusion. The ionic flux generated by the formation of an electric field is the second component, and the advection of ionic species by the fluid velocity field is the third component. The first and third components are the most important contributors to ionic flux in the case of the SANE sensor because they cause the diffusion and convection of the ionic species in the electrolyte. Both of these components are coupled to the second component of electrokinetic flow (migration in electric field) and the related computational module in the COMSOL will be discussed in the next section.

The continuity and momentum equations (Navier-Stokes) can be used to define a Newtonian fluid in an isothermal condition and by coupling these equations with the computational fluid dynamics technique in the entire computational domain of the SANE sensor. For simulating fluid motion, the Reynolds number *Re* = *ρvL/μ* needs to be computed. This is a dimensionless quantity which distinguishes the laminar from the turbulent flow regime, where *v* and *L* are the flow velocity and length scale of the nanopore in the sensor (~160 nm). In the present study, since fluid velocity is very low, *Re* is estimated to be ~0.0001, placing these simulations in the laminar flow regime. Also, because the advective term in the NSE can be neglected when viscous forces are greater than inertial forces, which are negligible here, and under steady-state conditions for fluid flow, we have a simplified momentum equation valid for low *Re* values, known as Creeping flow:

(2–5) $$\nabla^{2}u=-\nabla p$$

The electrokinetic transport within the nanopores is governed by the combination of eqs. 2–1, 2–4, and 2–5.

To compute the AC electric field eq. (AE) in environments where both AC voltage bias at various frequencies and a laser light field are present, and magnetic effects are negligible, we focus on the behavior of the electric field in response to these conditions. The AC electric field (*E*) in such scenarios is influenced by the alternating nature of the applied voltage, leading to a time-varying electric field that interacts with the medium’s permittivity (*ϵ*) and conductivity (*σ*). In the absence of free charges and considering a non-magnetic medium, the equation simplifying the electric field’s response under an AC regime can be derived from Maxwell’s eqs. (ME). The modified form of the wave equation, also known as the Helmholtz equation, is used to describe the electric field’s behavior in materials subjected to an AC electric field:

(2–6) $$\nabla^{2}E-\gamma^{2}\text{E}=0$$

where γ^2^= *jω*μϵ – *σ* represents the complex propagation constant, with *ω* as the angular frequency of the AC electric field, *μ* as the permeability of the medium (simplified to the permeability of free space *μ*0 when magnetic effects are neglected), *ϵ* as the permittivity of the medium, and *σ* as the conductivity. This equation accounts for both the displacement and conductive currents within the medium, influenced by the AC electric field.

To calculate the light field distribution created by light focused onto the sensor, Maxwell’s equations are solved. The interaction of the light field with the material is described by a form of Maxwell’s equations that emphasizes the electric field’s behavior in the presence of a light source. This takes into account the material’s properties such as permittivity (*ε*_0_), permeability (μ_r_), and conductivity (*σ*), and how these are influenced by the laser’s frequency (*ω*) and intensity. The equation for the light field distribution, considering the electric field (*E*) as influenced by a focused laser, is given by:

(2–7) $$\nabla \times {\mu_{r}}^{-1}(\nabla \times E)-{k_{0}}^{2}\left(\varepsilon_{r}-\frac{j\sigma }{\omega \varepsilon_{0}}\right)E=0$$

where *E* is the electric field amplitude, *ε*_0_ is the permittivity of vacuum, *j* denotes the current density, *ω* is the angular frequency, *σ* is the electrical conductivity, *μ*_*r*_ is the relative permeability of the material and *ε*_*r*_ is the relative permittivity. This equation effectively captures the complex interplay between the electric field and the material’s properties in the presence of a laser light field, allowing for the analysis and simulation of the sensor’s response to these combined influences.

The final equation is the convective heat transfer, which for the steady-state domain and can be represented by the heat equation:

(2–8) $$\rho {∁}_{p}\left(\frac{\partial T}{\partial t}+u.\nabla T\right)+(\nabla q)=\nabla u+Q$$

where *ρ* is the fluid density, ${∁}_{p}$ is the specific thermal capacity, *u* is the fluid velocity, *q* is the heat flux by conduction and *Q* is the heat source. This equation effectively captures the dynamics of heat transfer by convection within the simulation domain, incorporating both the transport of energy due to fluid motion and the diffusion of heat. The interplay between Eqs. (2–7) and (2–8) dictates the temperature distribution arising from the interaction of a light beam with the sensor, including effects of illumination and absorption.

### 2.3. Finite element modeling of multi-frequency phase shift and conductance in COMSOL

#### 2.3.1. Computational module integration

Building on the governing equations outlined above, we conducted simulations with COMSOL Multiphysics (version 5.6, Natick, MA) to analyze the phase shift and conductance responses of the SANE sensor under the combined influence of AC and DC electric fields, as well as their interactions with laser-induced light fields, through the plasmonic coupling of the latter. The plasmonic effect was simulated by incorporating power dissipation density as an input parameter to the AC electrical field module. This approach was adopted to effectively model this interaction despite the limitations in directly coupling the optical wave module with the electrical current in COMSOL. We utilized a 2D simulation domain that consisted of two ionic liquid reservoirs linked by two structures (DNH gap in the Au layer and the ssNP in the Si_3_N_4_ layer), each measuring 30 nm in width, as depicted in Fig. 1(a). For all computations, a *‘Physics Controlled Mesh*’ with the ‘*Finer*’ setting was selected in the software’s mesh generation section to accurately depict the subtle changes in potential, electric fields, ionic concentration, and velocity around the charged membrane surface. Also, quadratic triangular elements were utilized in the finite element calculations to enhance precision.

**Fig. 2.**
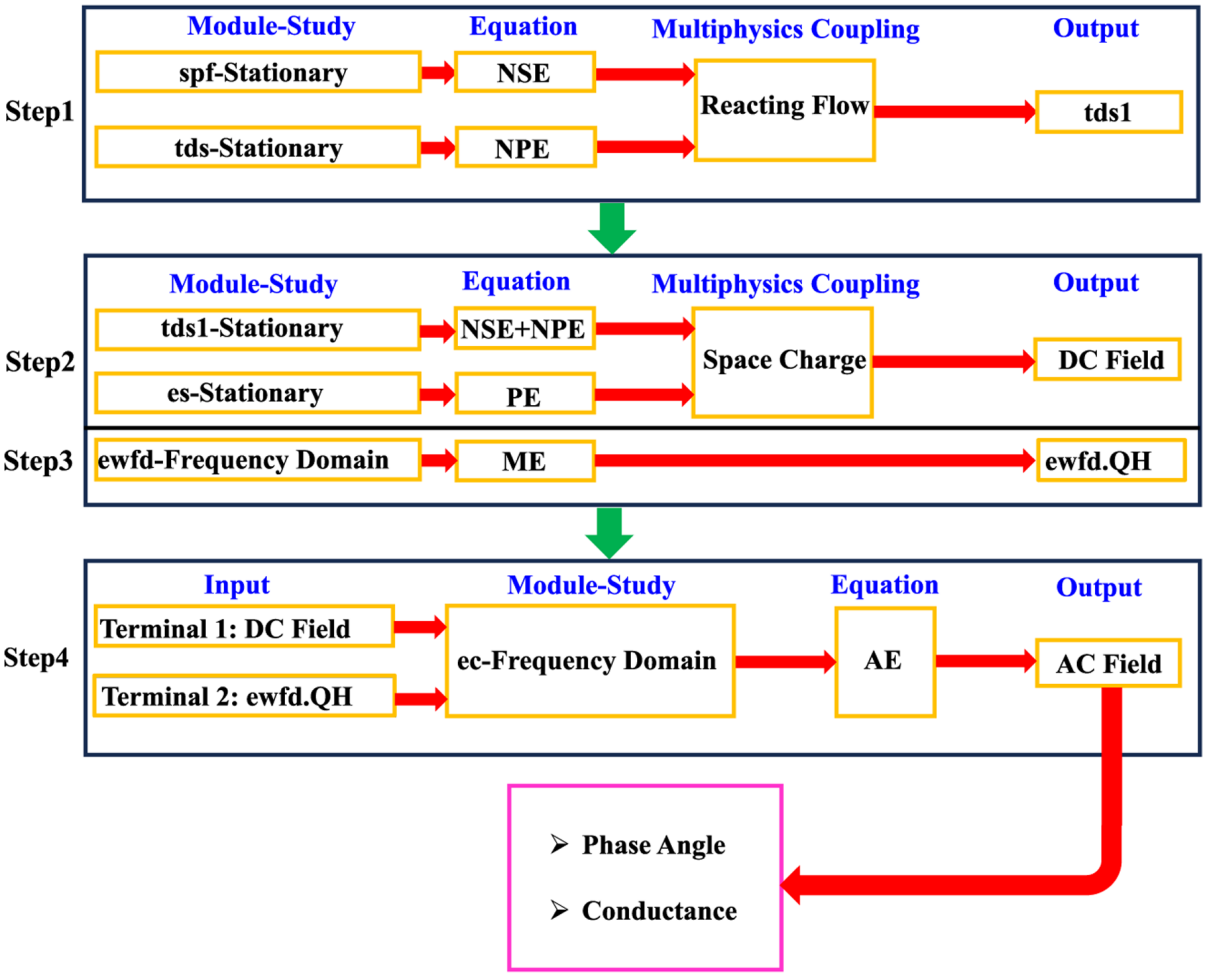
Flowchart illustrating the sequential coupling of computational modules in the COMSOL simulations. Step 1 encompasses the stationary studies of the *spf* and *tds* modules, resulting in the *tds1* output via Multiphysics coupling through *Reacting Flow*. Step 2 details the stationary simulation of the *tds1* module combined with the *es* module, leading to the DC Field output via *Space Charge* Multiphysics coupling. In Step 3, the frequency domain study of the *ewfd* module generates *ewfd.QH*. Finally, Step 4 represents the frequency domain study in the *ec* module, taking inputs from both the DC Field and *ewfd.QH* to produce the AC Field, from which Phase Angle and Conductance are derived. NPE: Nernst plank Equation; NSE: Navier-Stokes Equation; PE: Poisson Equation; ME: Maxwell Equation; AE: AC Electric Field Equation.

The simulation began with the NPE (Eq. 2–4), which computed the dynamics of ions in the electrolyte, achieved through the *Transport of Diluted Species* module (*tds*). Subsequently, the NSE (Eq. 2–5) defined the movement of water, facilitated by the *Creeping Flow* module (*spf*). Following this, the *Reacting Flow* as a Multiphysics coupling feature was employed to link the *tds* and *spf* modules (Fig. 2, step 1). The output of this coupling was termed *tds1*, wherein the movement and diffusion of the ions were calculated. The PE (Eq. 2–1) was used to describe the electric field distribution throughout the simulation volume using the Electrostatics COMSOL module (*es*). Subsequently, the *Space Charge* was employed as a Multiphysics coupling feature to link *tds1* and ES (Fig. 2, step 2), with the output of this coupling being the DC electric field. Thus, up to this point, the *spf*, *tds*, and *es* modules had been coupled, resulting in the DC electric field as the final output. Additionally, the ME (Eq. 2–7) was used to describe the light field distribution, achieved through the *Electromagnetic Waves, Frequency Domain* module (*ewfd*). Then, the power dissipation density (*ewfd.QH*), a predefined result of the *ewfd* module, was applied as an input terminal for the next step in the calculation (Fig. 2, step 3).

**Fig. 3.**
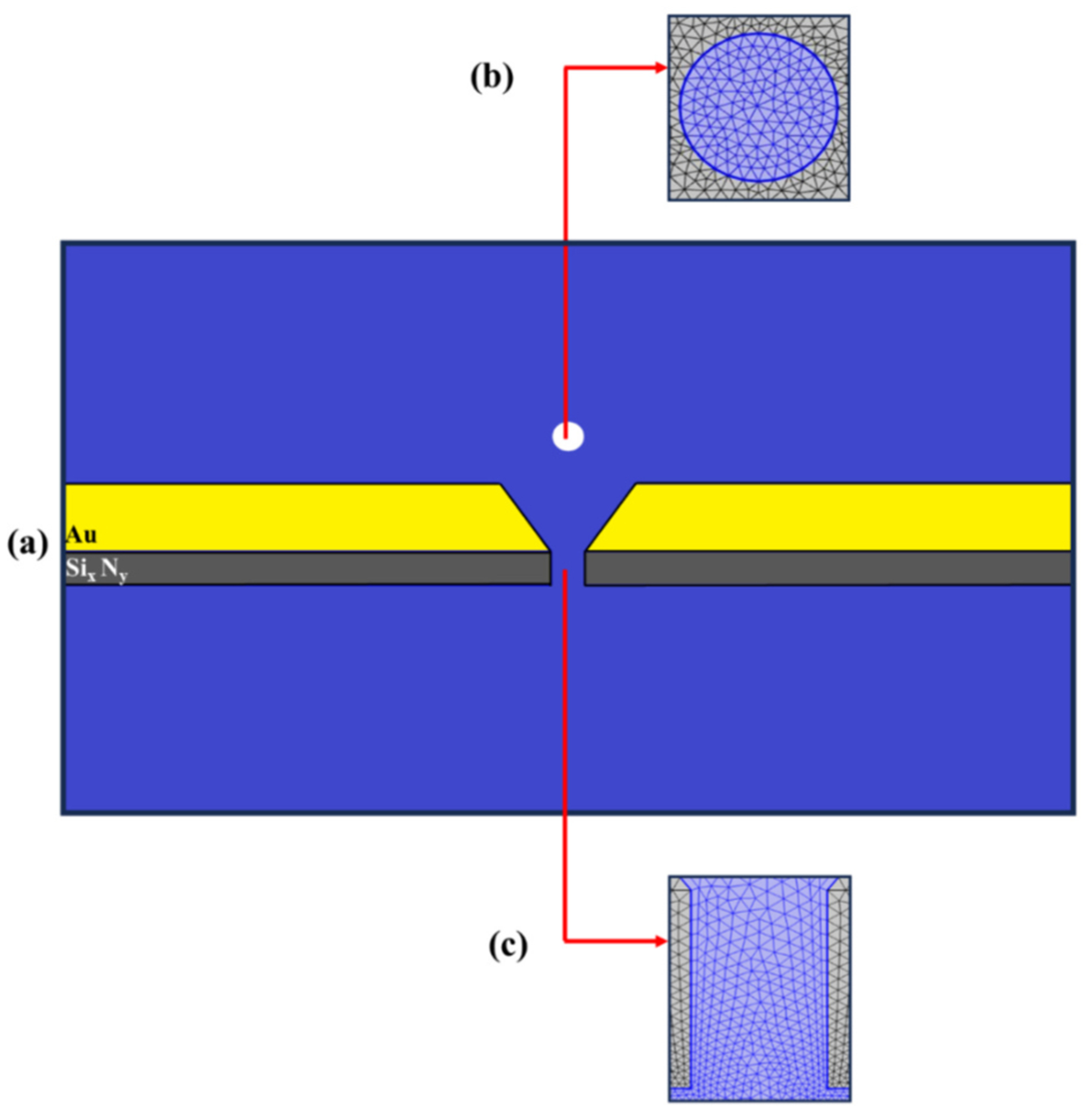
The computational domain of the SANE sensor is depicted. The yellow, gray, blue and white indicate the Au layer containing the DNH gap, the SiN layer with the ssNP, the electrolyte, and the nanoparticle, respectively. The mesh elements are shown in the computational domain inside the nanoparticle (b) and inside the nanopore region (c), magnified and viewed in cross in cross-section. Near domain sharp corners and near boundaries, such as the regions surrounding the nanoparticle, the mesh is finer, while in the far-field domain it becomes coarser.

Finally, the AE (Eq. 2–6) was solved using the *Electric Current* (*ec*) module to accurately characterize the AC electric field within the same simulation domain. Two terminals were established as inputs for the *ec* module (Fig. 2, step 4, Terminals 1 and 2) to calculate the AC electric field, incorporating all modules in the simulation. This process was followed by integrating the cumulative impacts of these modules (*spf*, *tds*, *es*) as a DC electric field, along with the effects of *ewfd.QH* as a laser, into the *Electric Current* module (Fig. 2, step 4), where the AE was solved. This integration was crucial, serving as a key input to ensure the influences of all these phenomena were included in the final analysis results. Ultimately, a stationary study was conducted to solve the NPE, NSE, and PE, (Fig. 3 steps 1,2), while a frequency domain study was specifically designed to solve the ME and AE (Fig. 3, steps 3, 4), with the added objective of capturing the AC electric field at pre-defined frequencies. Lastly, with the AC electric field determined, the conductance and phase angle were computed. Phase was calculated with the ‘arg’ function. Nanopore conductance (*I/V)* was calculated as the net ion current (*I*) crossing the nanopore as ion flux multiplied by the pore area, divided by the known command voltage (*V*). The current was computed using the formula *((tds1. tflux_C1* + *tds1.tflux_C2)* × *F),* where *C1* and *C2* represent the ions *K*+ and *Cl*−, respectively, and *F* stands for the Faraday constant. The term *tds1.tflux* refers to the total ionic flux, a parameter obtained from the *Transport of Diluted Species* module.

It is important to note that a practical limitation encountered in the computational aspect of this study was the software’s inability to fully integrate all relevant physical phenomena. Although COMSOL Multi-physics is capable of solving the equations describing the influence of the externally applied voltage bias on the sensor, the movement of fluid flow, and the migration of KCl ionic liquid in a fully coupled manner, it does not support the integration of Maxwell’s equations with these phenomena. This omission is significant as the light field plays a crucial role in the sensor’s functionality. To address this limitation, we approximated the impact of the light field in our simulations by calculating the power dissipation density it induced and used this calculation as an input for subsequent steps.

#### 2.3.2. Boundary conditions

For the boundary condition, in the *Electrostatic* module (*es*) for solving the PE, a surface charge density for the nanopore wall and nanoparticle surface were created (Table 2) and an electrical potential and ground were assigned at the bottom and top borders respectively (*trans* and *cis*). In the *Creeping Flow* module (*spf*), a boundary condition of *P* = 1 atm (standard atmospheric pressure) was applied to both the inlet and outlet on the top and bottom surfaces, respectively, for solving the NSE [49]. Across all other solid interfaces, a no-slip velocity boundary condition was implemented to address the NSE. The electroosmotic velocity boundary condition was set for the nanopore wall to facilitate the calculation of the electroosmotic velocity field, since there is a Debye layer and electroosmotic phenomena present.

For the *Transport of Dilute Species* module, inflow and outflow boundary conditions were established at the bottom and top edges (the *trans* and *cis* sides) with a KCl concentration set to 0.3 M, to address the NPEs. Additionally, a zero (normal) electromigration and diffusion flux was applied to all other solid interfaces [50]. Furthermore, ‘*Convection*’ and *‘Migration in Electric Field*’ were chosen as the transport mechanisms within the transport of dilute species module. The diffusion coefficient and concentration for K+ and Cl− ions were assigned in the *Transport of Diluted Species* module (*tds*, Table 2).

In the *Electromagnetic Wave Frequency Domain* module for addressing the ME, a perfectly matched layer (PML) was implemented at the top and bottom surfaces to eliminate back-scattering at the external boundaries, and a perfect electrical conductor was presumed for the side perimeters of the Au layer. In the *Electrical Current* (*ec*) module for solving the AE, a boundary current source for nanoparticle surface were created. This source was calculated through the formula “*ϵ* × 2 × *π* × frequency×*E*”, allowing for a precise representation of the current density’s impact on the surrounding medium. Then, an electrical potential and ground were assigned at the bottom and top borders respectively (*trans* and *cis*). Setting the electric potential border completed all requirements for performing AC frequency simulations spanning 10–100 kHz.

### 2.4. SANE sensor fabrication

**Fig. 4.**
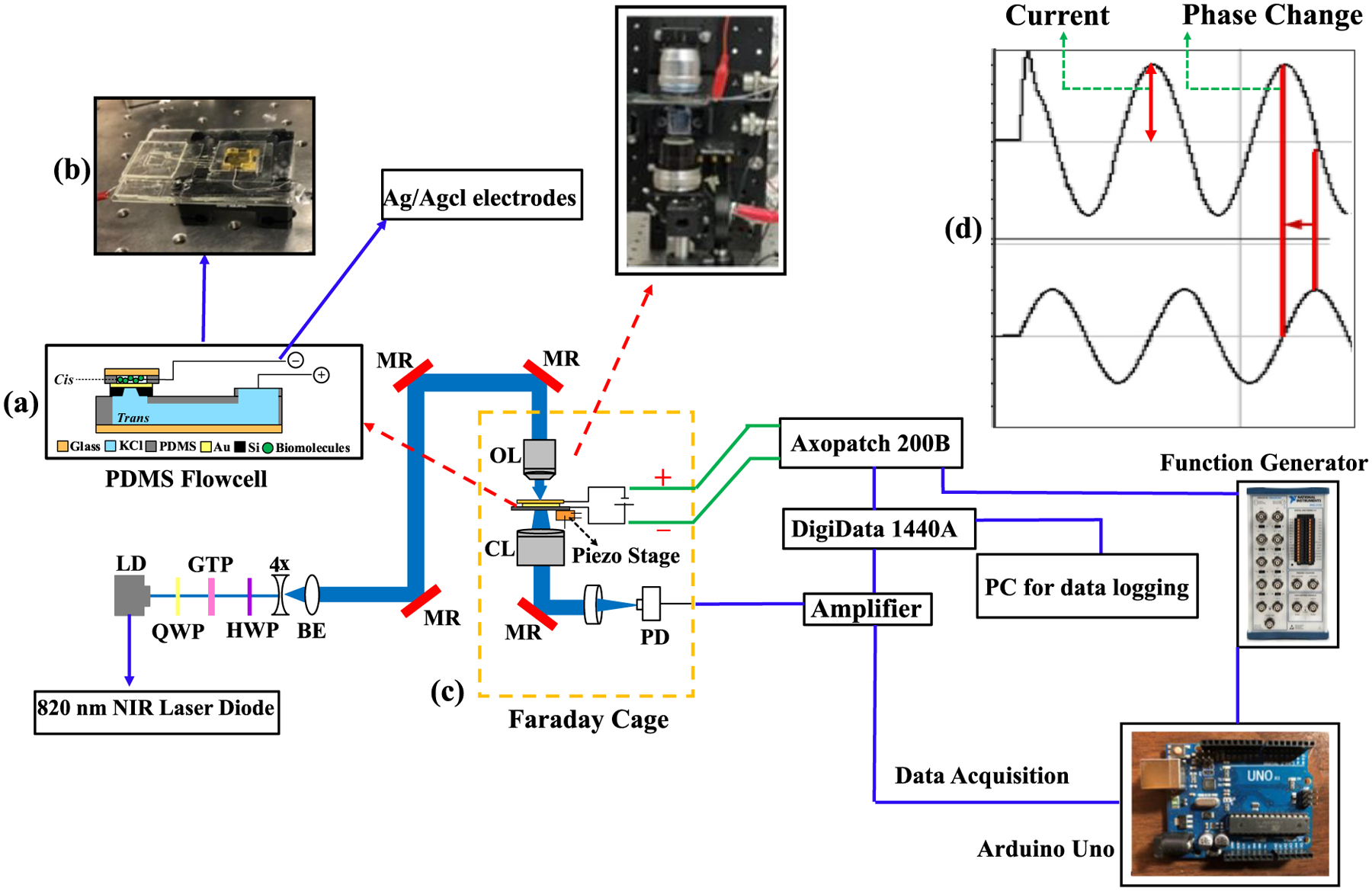
Experimental setup including flow cell, optical setup, and data acquisition equipment. (a) PDMS flow cell cross-sectional view with the SANE sensor. (b) Image of prepared PDMS flow cell with SANE chip ready for placement on piezo-controlled stage. (c) Optical setup with PDMS flow cell placement and measurement instruments. LD: laser diode, QWP: quarter wave plate, GTP: Glan-Thompson polarizer, HWP: half wave plate, 4× BE: 4× beam expander, MR: mirror, OL: Carl Zeiss 1.3 N.A. 63× objective lens, CL: condenser lens, PD: photodiode. The Arduino Uno and Agilent function generator were added to the experimental setup to provide optical-based triggering for the function generator and AC pulsed oscillation command signals to the Axopatch 200B. (d) Example schematic of the phase shift between the command AC voltage (bottom row) and detector response (top row). Conductance was calculated as the inverse of impedance = *I/V*, where the current *I* was the sensor current AC amplitude.

The procedure for fabricating the SANE sensor has previously been documented [46]. In summary, the sensors were created on clean 4-in. silicon (Si) wafers with a 500 nm silicon dioxide (SiO_2_) layer formed on top through thermal oxidation. A 60 nm layer of non-stoichiometric silicon nitride (Si_x_N_y_) was then deposited using low-pressure chemical vapor deposition. On the wafer backside, a grid pattern was applied using a darkfield mask (positive photoresist S1813) to divide the wafer into individual chips measuring 15 mm × 15 mm. The mask also defined a square window of 786 μm on each side, where the Si_x_N_y_ layer was etched away using deep reactive ion etching with tertrafluoromethane (CF4) gas at a rate of 1 nm/min. Subsequently, the underlying SiO_2_ layer was etched using a 6:1 buffered hydrofluoric (BHF) acid until reaching the underlying Si layer. The backside was then subjected to an anisotropic etching process using a 22 % tetramethylammonium hydroxide (TMAH) solution at 90 °C to create a 100 μm window, leaving the overlying Si_x_N_y_/SiO_2_ layers from the front side suspended. On the front side of the wafer, a 100 nm thick layer of gold (Au) with a 5 nm chromium (Cr) adhesion layer was deposited using the e-beam evaporation method at a rate of 0.1 nm/s. Alignment markers for focused ion beam (FIB) milling were patterned on this Au layer using photolithography, and the Au and Cr layers over the marker positions were etched using specific wet etchants. A thick layer of photoresist was applied as a protective coating, and then the wafer was diced into individual chips. Each chip underwent an acetone rinse to remove the photoresist layer, and the underlying SiO_2_ layer was eliminated using a 6:1 BHF solution. The individual chips were placed in a gas field ion source FIB (CNMS, Oak Ridge National Laboratory, Oak Ridge, TN), where the DNH nanostructures were milled through the Au layer using a Ne ion beam, and the nanopore was created in the Si_x_N_y_ membrane at the center of the DNH structure [see Fig. 4(c)], using a He ion beam. The typical dimensions for the DNH structures utilized in this study were 100 nm diameter circles that intersected to create tapered edges with 15 %–20 % slope, converging towards a 30 nm diameter pore located in the middle of the structure.

### 2.5. Experimental setup and AC data acquisition

Figure 4(c) depicts the experimental setup in schematic form. In brief, a laser diode (820 nm, L820P200, Thorlabs) emitted a collimated beam, which was transformed into a circularly polarized 2 mm diameter beam using an aspheric lens and a quarter-wave plate (QWP) (WPQ05M, Thorlabs). A Glan-Thompson linear polarizer (GTH10M, Thorlabs) and an adjustable half-wave plate (HWP) (WPH05M, Thorlabs) were utilized to select the optimal linear polarization aligned with the short axis of the DNH on each chip, enabling the optimal excitation of wedge plasmons. The beam expander (4×, Newport) ensured that the back aperture of a 63× oil immersion objective lens (NA = 1.2, Zeiss C-Apochromat) was fully filled through a periscope. Precise positioning of the laser beam at the center of the DNH was achieved by employing the alignment markers and adjusting the controls of a piezo stage (MDT6938, Thorlabs) that held the chip. The sensor was enclosed within a transparent polydimethylsiloxane (PDMS) flow cell, fabricated according to our previous work [52], with a coverslip placed on top to focus light onto the center of the DNH on each chip. The transmitted light was collected using a condenser lens and subsequently directed onto a photodiode (PDA36A, Thorlabs). The PDMS chip holder [Fig. 4(b)] featured a *cis* chamber where nanoparticle solutions mixed with potassium chloride (KCl) were dispensed, while the *trans* side contained only KCl solution of the same molarity (0.3 M).

The optical data was collected by a photodiode that recorded the transmitted light intensity through the sensor, which increased in a stepwise manner when a nanoparticle was trapped and then decreased when it translocated through the sensor. Electrical data consisting of a positive current spike when the nanoparticle entered the optical trap, current fluctuations while the particle stayed in the trap, and a negative spike when the nanoparticle escaped the trap and translocated through the immediately underlying nanopore, were collected synchronously using an Axopatch 200B system (Molecular Devices, San Jose, CA). Both optical and electrical data were digitized using an Axon Digidata 1440 ADC (Molecular Devices). For the AC measurements, the optical transmission data was also sent to an Arduino Uno (Adafruit Industries, New York, NY) programmed to perform edge detection, looking for an optical step change indicative of a trapping event, which was accompanied by a simultaneous current spike in the electrical data stream. The Arduino then sent a trigger to an Agilent 33250A Waveform/Function Generator (Agilent Technologies, Santa Clara, CA) that generated a user-defined pulse at a selected frequency and amplitude. The function generator set the command voltage for the Axopatch 200B, which was operated in voltage clamp mode. The ratio of measured current amplitude to command voltage i.e., conductance, was computed as a characteristic parameter of the nanoparticle response to AC voltage modulation over 10 sinusoidal cycles for a given frequency, which we will refer to as an AC burst event. Furthermore, fast-Fourier transform (FFT) analysis of the recorded AC signal responses enabled calculating the phase change while the particle was exposed to the AC burst inside the optical trap. The DC voltage was consistently kept on at 100 mV (−ve *cis* to +ve *trans*) for all experiments. A baseline AC response was established using a model cell reference block provided by the Axopatch 200B manufacturer for calibrating the system and isolating the sensor’s net response from the total system response observed.

Each sensor that was used in the AC measurements was also baselined using 40 μL of 0.3 M KCl solution at 7.4 pH. Baseline measurements were performed at 110 mV command voltage with one of the following AC frequencies superimposed: 20, 100, 1k, 10k and 80k Hz. Modulation at higher frequencies was not possible with our experimental setup due to a hard-wired Bessel filter in the Axopatch 200B system with a maximum cutoff frequency of 100 kHz. We therefore limited our maximum frequency measurement to 80 kHz to minimize any influence from the 100 kHz low-pass filter on the measured signals. The amplitude of the waveform at each frequency was set to ensure there was a high SNR but low enough to prevent the Axopatch 200B did not saturate while recording the current response. 20 Hz measurements were taken with a 10 V p-p signal and 1 kHz were generally collected at 50 mV peak-to-peak.

The Axopatch 200B front-switched command voltage port was used to connect to the function generator. This port reduced all signals by a factor of 20. Each frequency was set to pulse 5 times with 10 cycles each to enable testing the reproducibility of the response. In addition, signal decays recorded at the end of each frequency burst were analyzed to generate additional data types for the characterization of nanoparticles.

The 20 ± 4 nm SiO_2_ NPs (MEL0010, NanoComposix, zeta potential = −40 mV) were used as the analytes to study in this work because these had been well characterized in our previous work with DC SANE sensor measurements [6]. The SiO_2_ nanoparticles were tested at the same frequencies as the baseline measurement for comparison with the empty trap response and model cell (data acquisition hardware) response.

Once the data was collected, each pertinent AC event was noted for start and stop times as well as if it took place during a trapping event or not. The event parameters were imported into a MongoDB document database (MongoDB Inc., New York, NY) and then loaded into Matlab (MathWorks, Natick, MA) to first be processed for frequency response and then for decay response. The axon binary file (.abf) generated by the pCLAMP software (Molecular Devices) was trimmed according to the event times and an FFT was performed on the current response and command voltage of the .abf data as depicted in Fig. 3. The center frequency of the oscillation was determined by the FFT and was then used to identify the phase difference between the command voltage and the current response. The magnitudes of peak amplitude, with both current response and command voltage, were divided to calculate conductance of the sensor during the AC event. The phase shift at each frequency for the model cell was subtracted from the empty trap to determine the sensor specific phase change. The conductance at each frequency for the empty trap was divided by the model cell conductance to calculate the net conductance response of the sensor. In a similar manner, the phase change of the empty trap was subtracted from the corresponding measurement with the model cell, to derive the net sensor phase response.

### 2.6. Statistical comparison of simulation and experimental results

Computational results were compared with corresponding measurements performed at closely matched experimental conditions. Each experimental data point was the average of at least three repeat measurements at identical conditions, enabling to compute its mean and standard deviation. Simulations, in contrast, yielded values without any variance. Computational predictions were subtracted from the corresponding experiential measurements and the resulting data were tested by a one-sample *t*-test, for statistically significant differences from zero (*p* < 0.05). This approach tested how closely our simulations matched experimental measurements.

## 3. Results

**Fig. 5.**
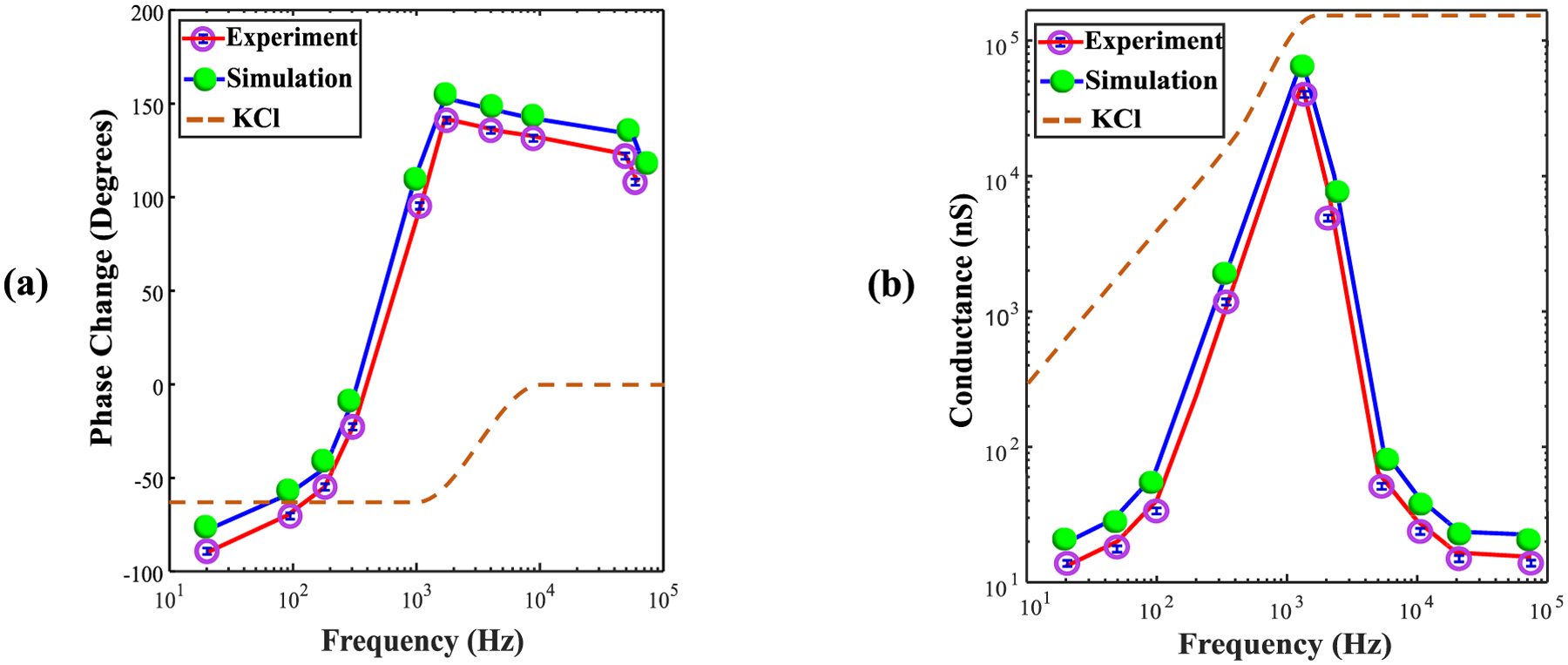
Comparison of experimental and simulation results for the frequency response of the empty SANE sensor for (a) phase shift, and (b) conductance as a function of AC frequency. In both panels, the purple markers represent experimental data points, and the green markers indicate simulation data. The blue error bars on the experimental data points represent the standard deviation from three independent measurements. Each panel also shows corresponding simulation data for a pure 0.3 M KCl solution (orange dashed lines) for comparison.

A comparison of the results of baseline experiments with the empty SANE sensor at 0.3 M KCl against simulations with parameters matching the experimental conditions are presented in Fig. 5. Significant phase delay and conductance changes were recorded as a function of AC frequency. After subtracting the model cell phase response i.e., the data collection instrumentation response, from that of the empty sensor measurements, the net sensor phase dependence on AC frequency was calculated. This sensor-induced phase change showed a rapid increase up to 1–2 kHz and then rolled off with a smaller slope, down to the highest recorded frequency of 80 kHz [Fig. 5(a)]. In addition, the model cell conductance response was divided out from the empty sensor measurements to deduce the relative conductance as a function of AC frequency. Relative conductance showed a peak in the 1–2 kHz range, and then dropped precipitously for frequencies above about 5 kHz [Fig. 5(b)].

The AC frequency response of the empty SANE sensor was also computed by COMSOL and compared with corresponding experimental results. As demonstrated in Fig. 5(a), the simulations predicted a peak phase shift of 149.2° at 1–2 kHz that was not far from the 145.3° recorded experimentally. Beyond this peak, both experimental and simulated data exhibit a declining trend in phase shift at higher frequencies. Fig. 5(b) compares conductance results between simulations and experiment as a function of frequency. A prominent conductance peak is seen near 2 kHz, where the simulations predicted a conductance of 42e4 nS versus 41e4 nS recorded experimentally. After the peak, a declining trend in conductance is observed as frequency increases from 10 kHz to 80 kHz, for both experimental data and simulations.

The AC impedance properties of pure 0.3 M KCl ionic solution, in the absence of a sensor, were also simulated to help determine the relative contribution of the solvent to the observed data trends. The results for phase shift [Fig. 5(a)] and conductance [Fig. 5(b)] for KCl solution also peak in the vicinity of 1 kHz, which suggests that solvent did play a role in determining the sensor sensitivity profile, although the overall profiles were qualitatively different and more likely to have been driven by the sensor materials and structure.

**Fig. 6.**
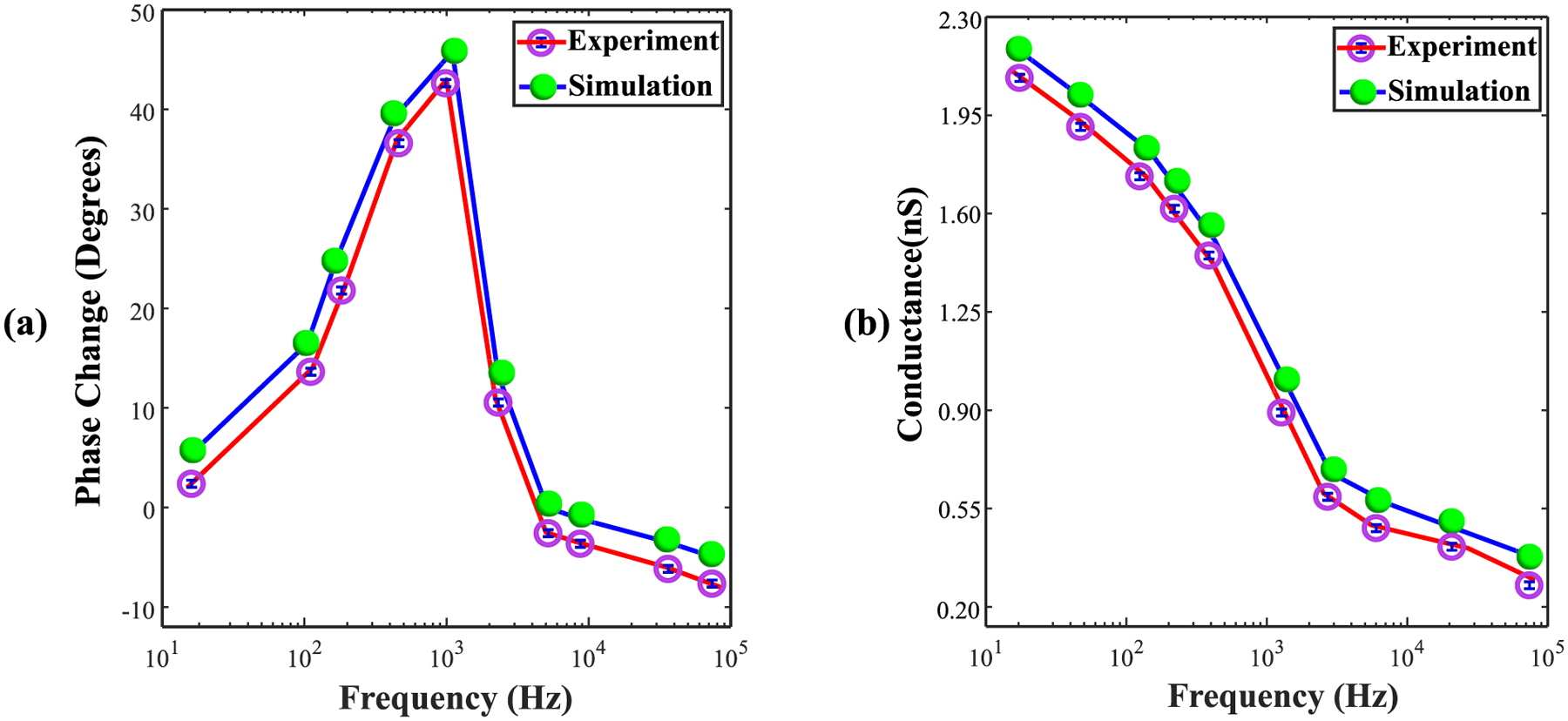
Comparison of experimental and simulation results for the frequency response of the SANE sensor with a 20 nm SiO_2_ particle trapped at its center. (a) phase shift and, (b) conductance as a function of AC frequency. In both panels, the purple markers represent experimental data points, while the green markers indicate simulation data. The blue error bars on the experimental data points represent the standard deviation from three independent measurements.

Next, AC sensing experiments with SiO_2_ 20 nm nanoparticles were performed at frequencies ranging from 20 Hz to 80 kHz. To minimize multi-particle interactions near the optical trap, experiments were conducted using an ultra-low concentration (1 fM). These results were then compared with the empty SANE sensor response in terms of both phase change and conductance. Fig. 6(a) highlights the phase shift response in the presence of nanoparticles, with the most significant shift occurring in the 1–2 kHz range for both experimental and computational results. The ratio of conductance between sensor signals, with and without an SiO_2_ nanoparticle trapped, peaks at low frequencies and decreases at higher frequencies. This conductance ratio also shows an inflexion point, with a reduced slope and therefore a reduced differential sensitivity above the 1–2 kHz range [Fig. 6(b)].

Comparisons of phase and conductance in Figs. 5 and 6 indicates some common features as well as significant differences in the frequency behavior of a nanoparticle-loaded versus an empty sensor. To help understand the underlying physical mechanisms that drive these patterns additional COMSOL simulations were performed, each focusing on a specific question.

**Fig. 7.**
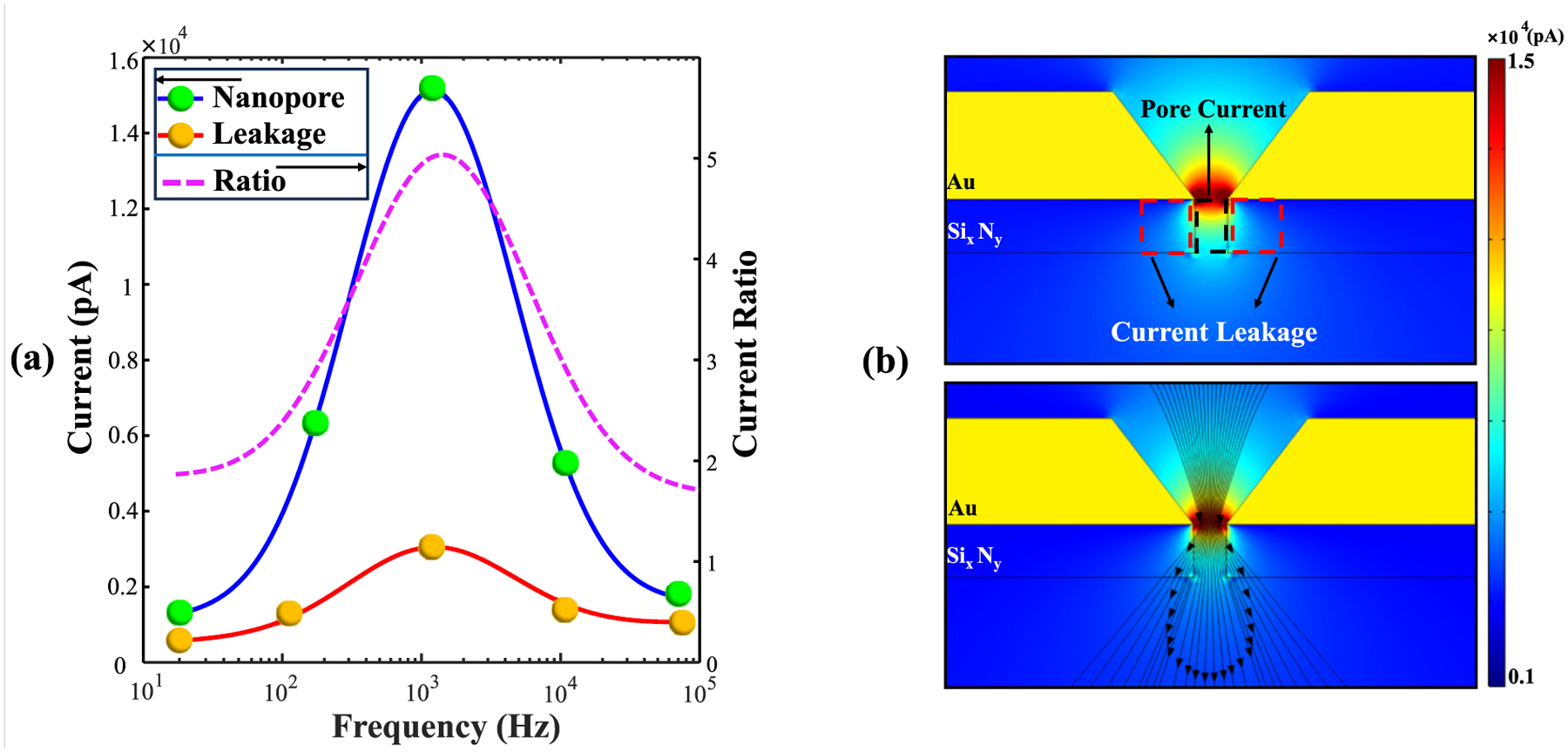
(a) Amount of current exiting the nanopore volume (blue curve) and leakage current exiting through the Si_x_N_y_ layer (red curve). Nanopore-to-leakage current is also shown (pink dashed curve, right *y*-axis). (b) The colour map visualizes the regions where current leakage occurs, outlined by red dashed lines. The streamlines in (c) demonstrate the directionality of current penetration into the nanopore and Si_x_N_y_ layer at 1 kHz.

The first question that we explored was whether leakage current through the solid materials of the sensor increased with increasing AC frequency, diverting useful sensing current away from the nanopore volume. The simulations indicated that since Au is a conductor, it shields the incoming electric field and therefore the only possibility of leakage occurs when AC current reaches the underlying dielectric Si_x_N_y_ layer (Fig. 7). The blue and red curves in Fig. 7(a) show the nanopore and leakage currents, respectively. While both currents peak near 1 kHz, the ratio of nanopore-to-leakage current is in the ~5–6 range near that peak and in the ~2 range at lower and higher frequencies. In Fig. 7(b), the area of current leakage is discernible, highlighted by red-dashed rectangles. To further clarify what the current distribution looks like at 1 kHz, Fig. 7(c) shows current streamlines. This figure shows that most of the current travels, as intended, through the nanopore and only a few current streamlines exit sideways through the Si_x_N_y_ layer and then into the KCl solution on the *trans* side.

**Fig. 8.**
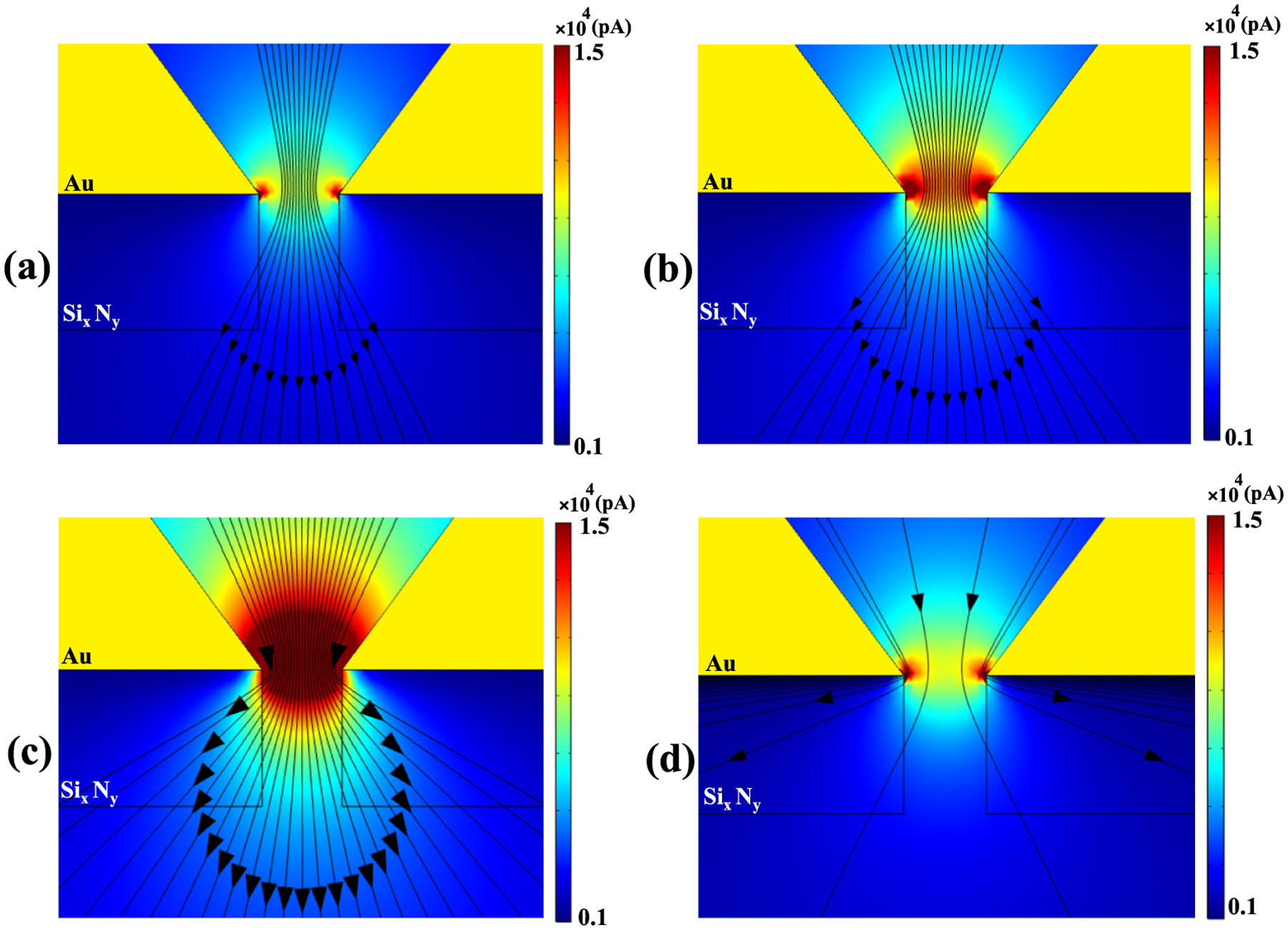
Current magnitude (colour map) and streamlines (constant current amplitude contours) under a 110 mV DC voltage with 110 mV AC voltage superimposed for the empty SANE sensor for AC frequencies of (a) 20 Hz, (b) 100 Hz, (c) 10 kHz, and (d) 80 kHz.

The effect of AC frequency on the current distribution through the nanopore sensor was then explored in more detail (Fig. 8). At the lower frequency of 20 Hz, the maximum current traverses predominantly through the center of the nanopore [Fig. 8(a)]. When frequency increases to 100 Hz, the constant current amplitude streamlines indicate charge transport occurring primarily through the center of the nanopore. At the same time, more streamlines are beginning to diverge sideways relative to 20 Hz, traveling through the nanopore, but also conducting more AC current through the Si_x_N_y_ layer [Fig. 8(b)]. At 1 kHz the current streamline density is highest [Fig. 7(c)]. At a frequency of 10 kHz, the current streamline distribution is less dense relative to 1 kHz [Fig. 8(c)], with increased sideways divergence and penetration through the Si_x_N_y_ layer. At the highest frequency tested of 80 kHz the streamline density reduced significantly and current conduction was mostly confined near the nanopore walls.

To gain some quantitative understanding of these qualitative observations, simulations were used to quantify current density near the Au surface as a function of AC frequency [Fig. 8(a)]. The motivation for these calculations was the observation that the simulated current density was highest near the surface of Au, rather than through the center of the nanopore, and more so with increasing AC frequency. This phenomenon is reminiscent of the known skin effect, where AC current tends to flow predominantly within a thin layer near the surface of a conductor at higher frequencies [62].

**Fig. 9.**
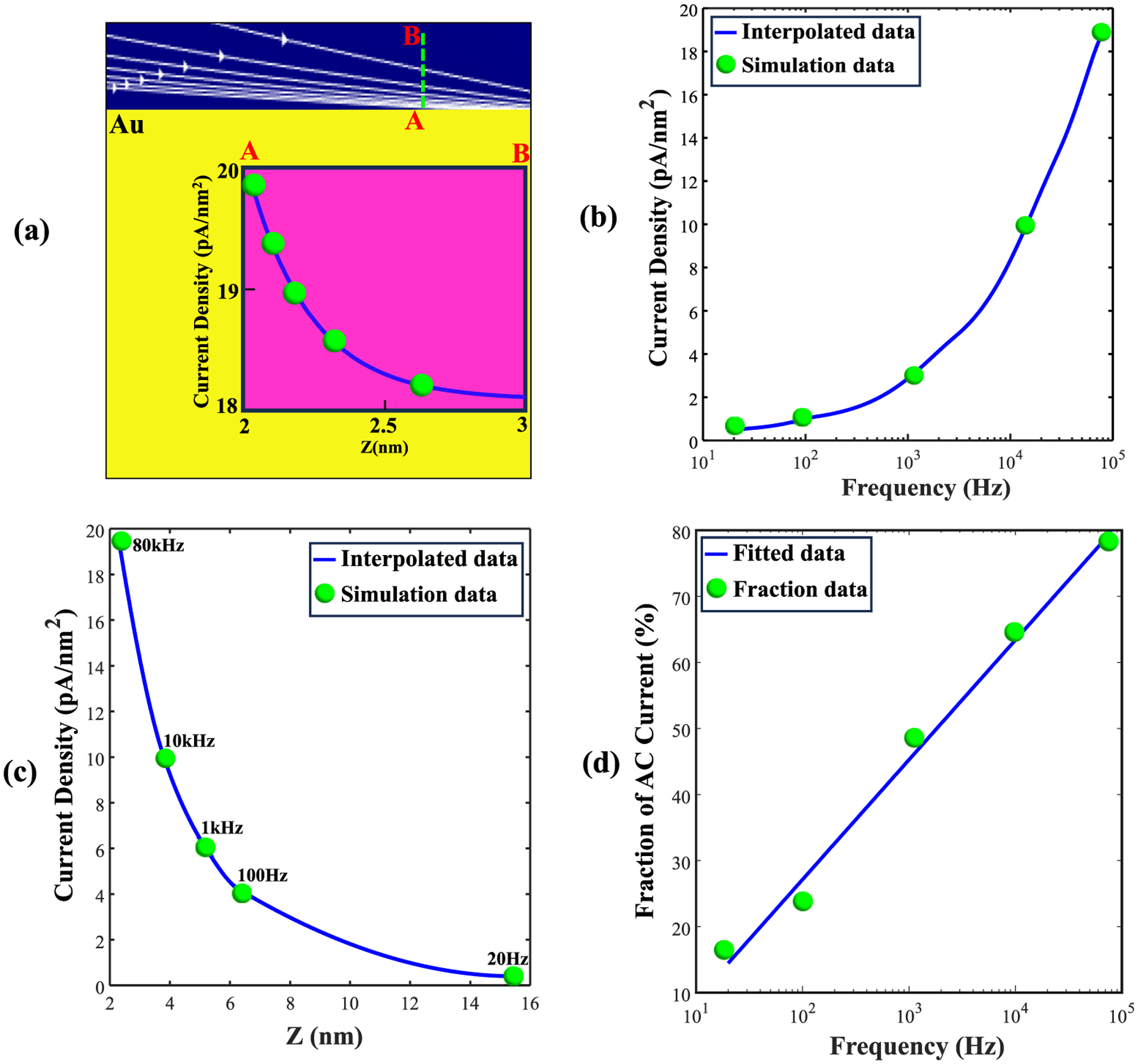
(a) The white streamlines indicate decreasing current density as a function of vertical distance above the Au layer, as also indicated by the plot of current density versus *z* between sample points A and B (inset). (b) Current density averaged over a 1/e distance from the current maximum occurring just above the Au surface, plotted as a function of AC frequency. The blue curve represents interpolated data, while green markers indicate computational data points. (c) Current density averaged over the 1/e thickness *ΔZ* versus AC frequency. (d) Ratio of the current in the 1/e-thick layer near the wall to the rest of the current in the pore.

First, it was verified that the KCl solution above the Au layer followed an exponential current density as a function of distance from the Au surface, to balance the skin current [Fig. 9(a)]. The 1/e decay value of current density profile in KCl was calculated for each AC frequency as a function of distance above the Au surface (*ΔZ*) and plotted in Fig. 9(b).

These calculations showed that current density increased with increasing AC frequency, but the current travelled through an ever thinner 1/e layer, which reduced the total amount conducted at higher frequencies. Although the skin-effect phenomenon does not hold away from conducting surfaces, the simulation streamlines indicated that as AC frequency increases, the current that was confined near the Au surface continued to mostly travel near the walls of the underlying nanopore also. Following up on this line of thinking, we calculated the fraction of current traveling through a 1/e-thick layer of KCl solution away from the nanopore walls versus the remaining, central, volume of the nanopore. The resulting current fraction flowing near the surface of the nanopore indeed became dominant at the highest frequency studied: 16.8 % (20 Hz), 22.3 % (100 Hz), 49.2 % (1 kHz), 64.2 % (10 kHz) 79.5 % (80 kHz). These simulation predictions are also consistent with the fact that pure KCl solution conductance reduces [Fig. 5(a)] while the Au-driven skin effect intensifies [Fig. 9(a)] with increasing AC frequency.

**Fig. 10.**
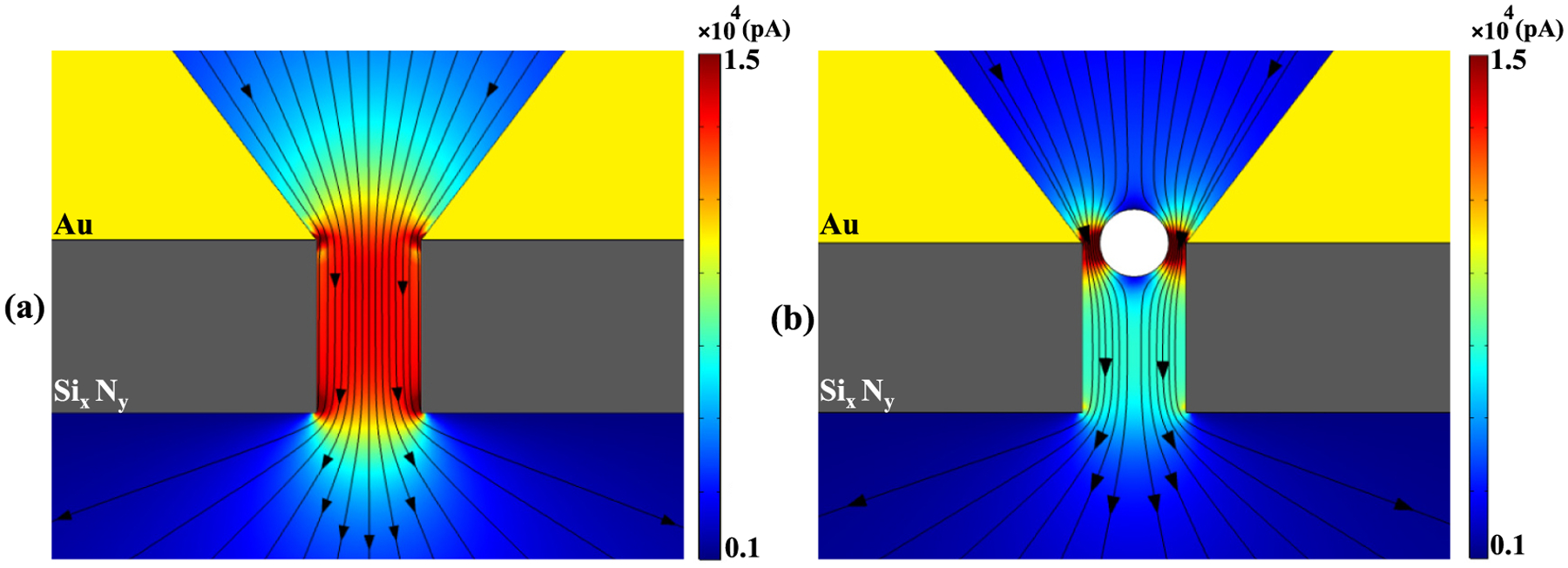
Current magnitude (colour map) and streamlines (constant current amplitude contours) under a 110 mV DC voltage: (a) for the empty SANE sensor, and (b) for a 20 nm SiO_2_ nanoparticle at the center of the sensor’s optical trap.

Next, we investigated how the presence of a 20 nm SiO_2_ nanoparticle affects the redistribution of current near the *cis* side of Si_x_N_y_ nanopore, first for DC and then for AC current at different frequencies. Fig. 10 illustrates the current distribution for a 110 mV DC field applied across the SANE sensor, with and without a particle trapped at its center.

In Fig. 10(a), the current streamlines are shown to traverse the entire cross-section of the nanopore in a relatively uniform manner, except for some attenuation occurring near the nanopore walls due to the counterions. On the other hand, Fig. 10(b) indicates that when a nanoparticle is introduced, the current streamlines bend around the particle, creating hotspots of current density in the regions near of the nanopore mouth. Furthermore, the current streamlines flowing through and exiting on the *trans* side in Fig. 10(b) suggest that the presence of the nanoparticle casts a ‘shadow’ and limits current conduction through the center of the nanopore.

**Fig. 11.**
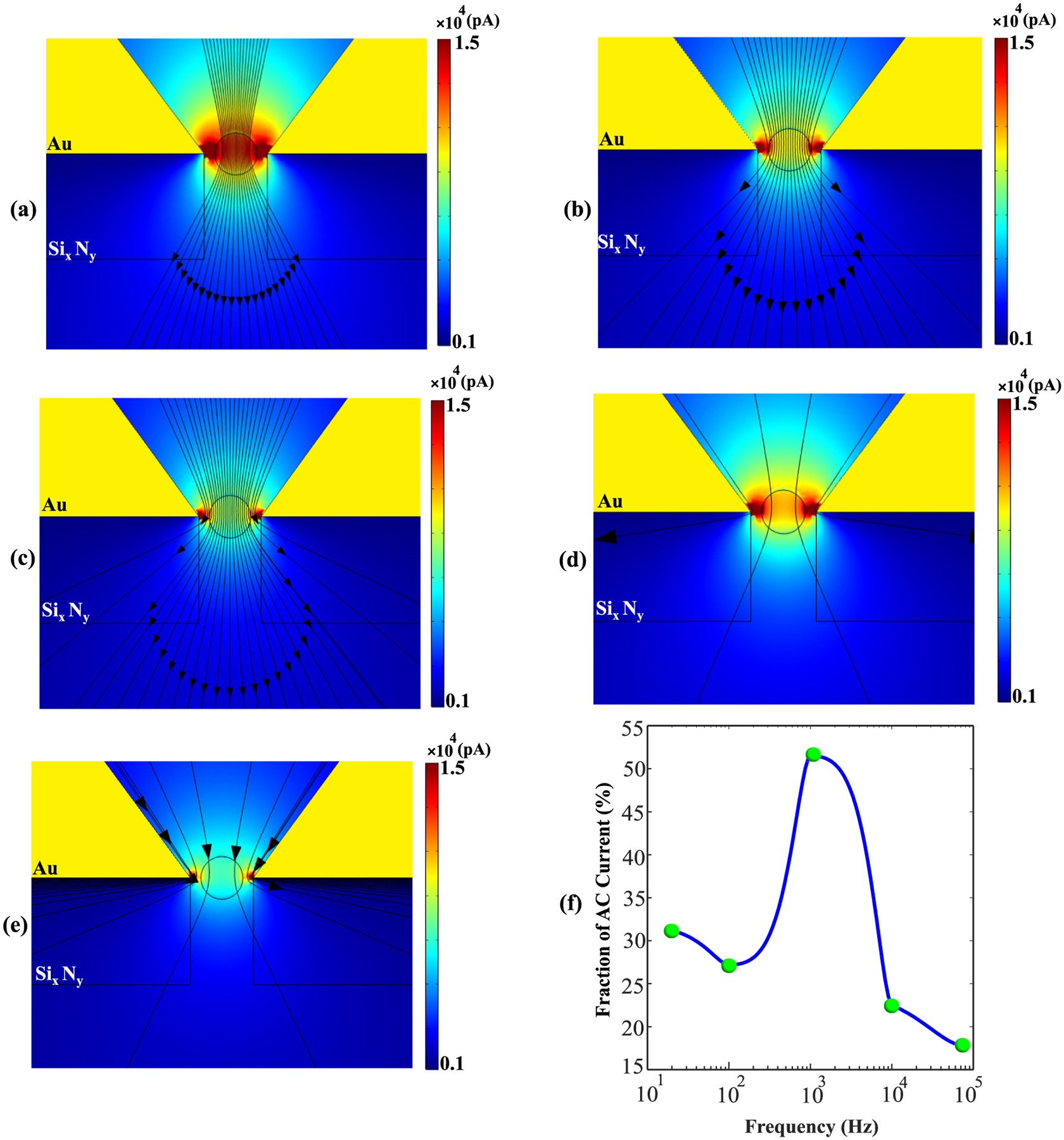
Current magnitude (colour map) and streamlines (constant current amplitude contours) under a 110 mV DC voltage with an amplitude of AC voltage superimposed for a 20 nm SiO_2_ nanoparticle at the center of the SANE sensor’s optical trap for AC frequencies of (a) 20 Hz, (b) 100 Hz, (c) 1 kHz, (d) 10 kHz and (e) 80 kHz. (f) Fraction of the AC current flowing through versus around the nanoparticle as a function of frequency.

Lastly, Fig. 11 shows the alterations in current distribution created at the SANE sensor when a nanoparticle is placed at the center of its optical trap and is subjected to different AC frequencies. At the lowest tested frequency of 20 Hz the current registers a maximum, which indicates that the dielectric nanoparticle provides a conduction channel for current to travel through, which was not available in the empty sensor scenario [Fig. 11(a)]. The streamlines indeed seem to travel preferentially through the center of the particle at 20 Hz and increasing the frequency to 100 Hz not only results in a slightly reduced nanopore current, overall, but also demonstrates that there is less current traveling through the particle and more around the particle [Fig. 11(b)]. At 1 kHz, contrary to the behavior of the empty sensor, the NP presents a semi-permeable barrier for the current, which seems to travel more around it than through it, while leakage through the Si_x_N_y_ layer also increases [Fig. 11(c)]. At the highest tested frequency of 80 kHz, the Au skin effect is the strongest and most of the current travels around the particle, significant leakage through the Si_x_N_y_ layer [Fig. 11(d)]. To verify these qualitative observations, we calculated the fraction of AC current passing (along the normal) through the nanoparticle versus around to obtain: 31.2 % (20 Hz), 27.2 % (100 Hz), 50.5 % (1 kHz), 22.5 % (10 kHz), 17.8 % (80 kHz) [Fig. 11(f)]. These results are consistent with other findings in this work, all pointing to the fact that the ~1 kHz region is the most sensitive operation point of this sensor.

## 4. Discussion

The main objective of this study was to enhance our understanding of the electrical properties of the SANE sensor when operated under AC voltage bias, in the presence of a 20 nm SiO_2_ nanoparticle trapped optically at its center, and for the empty sensor. Specifically, COMSOL Multiphysics simulations were employed to investigate the phase shift and conductance as a function of applied voltage frequency, up to near the highest frequency of 80 kHz allowed by the current experimental setup. To mitigate the massive computational load that solving this multi-physics problem in 3D entails, an approximation was made where the optical plasmonic wave structure was simulated in 3D, since the DNH is a resonant structure, but the remainder of the multi-physics problem was solved in 2D across a plane cutting through the center of the DNH’s long axis. Despite this significant simplification, the simulations performed well in approximating the patterns observed in the AC measurements. It should also be noted that when performing these comparisons, the AC phase and conductance measurements in Fig. 5 were baselined to measurements with a model cell, consisting of a 500 MΩ resistor, to factor out the effect of the recording device from the measurements.

Another noteworthy point is the SANE sensor’s phase shift behavior in the ~1 kHz region. Near that frequency, the sensor’s phase response is null, and the phase gradient is highest. AC conductance is also maximal at that frequency region, making this the most sensitive operational point of the sensor for the given experimental conditions. To help explain the observed sensor data, we also investigated the intrinsic AC conductance impedance of the 0.3 KCl solution, with no sensor present. As shown in Fig. 5, the KCl conductivity profile appears to have a very different frequency response relative to the SANE sensor, and remains flat in the 1 kHz region, making it a non-significant contributor to the sensor’s frequency-dependent sensitivity profile in that region.

Further comparisons were made of the conductance profile of the SANE sensor with a SiO_2_ nanoparticle trapped at its center and contrasted these results with the empty sensor ones. It was noted that the frequency of maximum sensitivity in phase shift, near 1 kHz, seen in Fig. 5(a) corresponds to the frequency of maximum phase shift in the presence of a nanoparticle [Fig. 6(a)]. Interestingly, the highest conductance through the sensor happened at low frequencies, but the frequency of maximum sensor sensitivity was near 1 kHz, where a point of inflexion was seen in the conductance curve with the current decreasing more slowly at higher frequencies [Fig. 6(b)]. In all, one could say that the presence of the SiO_2_ nanoparticle acted as a low-pass filter on the sensor’s conductance profile.

An additional point that merits attention is the presence of higher leakage current when performing AC measurements compared to DC ones, as DC currents are blocked by dielectric materials. The Au layer serves as an effective barrier to the electric field, directing AC current to travel preferentially through the nanopore since it insulates most of the underlying Si_x_N_y_ dielectric layer. However, AC voltage does permit some current penetration through the underlying Si_x_N_y_ dielectric layer in places where the Au DNH is milled (not modeled in this work) and right after current enters the pore (Figs. 7 and 8). We therefore used the COMSOL simulations to estimate the relative contributions of the current traveling through the nanopore volume versus the current leaking through the solid substrate surrounding the nanopore volume [Figs. 7 (b)-(c)], which would not contribute to useful analyte-related signal. Fig. 7(a) shows the results for the empty SANE sensor, which indicate that the highest values of both nanopore current as well as leakage current occur at the sensor’s most sensitive operational point of ~1 kHz. The highest ratio of useful nanopore current to leakage current is also highest at ~1 kHz [Fig. 7(a), right axis].

Observation of Fig. 7(a) generated a follow-up question on what the mechanism of lower leakage current at higher frequencies could be, even though the Si_x_N_y_ layer would be more conductive with increasing frequency. Fig. 8 presents a visual narrative of the evolution of current transport with increasing frequency. The panels of this figure indicate an increased angular divergence of the portion of the current traveling through the Si_x_N_y_ layer with increasing frequency, consistent with that dielectric material being more conductive. However, the density of streamlines for current also become sparser with increasing frequency, indicating less current conducting through the sensor overall. There are two possible contributions to this phenomenon. First, the conductivity of KCl decreases with increasing frequency [Fig. 5(a)]. Second, the Au layer of the sensor has a frequency-dependent skin effect in its conductance [63]. AC skin current induced in the Au layer travels ever closer to the Au surface with increasing frequency (increasing current density), but the absolute value of the total current in KCl driven by this effect reduces with increasing frequency.

The skin effect’s effect on KCl concentration near the Au surface, as a function of AC frequency, was computed using COMSOL and is shown in Fig. 9. We quantified current density through a KCl layer of 1/e thickness, relative to the maximum current just above the Au surface and averaged it over that thickness (current density). Fig. 9(a) shows that current density increases with increasing AC frequency, as the current layer thickness decreases exponentially [Fig. 9(b)].

Interestingly, when calculating the fraction of current traveling through the 1/e thickness away from the nanopore mouth versus the rest of the pore, one sees increasing dominance of the current fraction traveling near the pore walls with increasing AC frequency. These ratios indicate a central-to-peripheral shift in current distribution with increasing frequency, which indicates that analyte detection sensitivity is confined more strongly near the nanopore walls at higher AC frequencies.

In the presence of a trapped nanoparticle the conductance response of the SANE sensor to a DC voltage is in stark contrast to its response to AC voltages. Under a DC voltage bias, the current traveling through the empty nanopore is homogeneous in the nanopore, except near pore walls [Fig. 10(a)]. Importantly, there is no current leakage through the sensor materials in this scenario. In the presence of a trapped nanoparticle, the SiO_2_ dielectric material is impermeable to the DC current, which is forced to squeeze around it to reach the underlying nanopore [Fig. 10(b)]. Thus, the conductivity under a DC field is primarily governed by the physical obstructions within the nanopore and the electrochemical properties of the electrolyte, leading to a predictable and steady current flow that is less influenced by material frequency-dependent properties.

In the case of AC sensing with a nanoparticle trapped at the sensor, at frequencies up to ~1 kHz the dielectric permittivity of SiO_2_ allow the nanoparticle to have higher conductance than at higher frequencies [64]. In the latter case the dielectric permittivity of the nanoparticle is outpaced by the ability of the Au layer to drive current in the KCl solution through the skin effect, as discussed above. The result is that current now passes preferentially through the sides of the particle, near the walls of the nanopore, at higher frequencies. The fraction of AC current passing through the nanoparticle versus around it is consistent with the ~1 kHz region being the most sensitive operation point of this sensor, for the above-specified experimental conditions.

## 5. Conclusion

The simulations and experiments conducted in this study provide valuable insights into how AC sensing performed by the SANE sensor, can enable researchers to probe the interior of a nanoparticle to varying degrees by adjusting the AC voltage frequency applied across the sensor. In the next step of this work, we plan to use this method to probe the load inside biologically relevant nanoparticles intended for drug and gene delivery. Therefore, the results of this work provide a useful first baseline for future exploration of the utility of using AC SANE sensing as a means of probing nanoparticle loading efficiency in therapeutic preparations.

## Figures and Tables

**Table 1:** Optical and electrical properties for the sensor’s Au (100 nm thick) and Si_3_N_4_ (50 nm thick) layers for DC voltage and the AC voltage frequencies for which simulations and experiments were performed.

Material	Thickness (nm)	Optical properties	Electrical properties	
		Complex Refractive (830 nm) Index (n + ik)	Dielectric constant (*ɛ = ɛ*_1_ + *iɛ*_2_)	Conductivity
Au	100	0 = 0.16126 K = 5.1558	Drude model	Drude model
Si_3_N_4_	50	*n =* 1.99 k ~ 0	Lorentz model	~0.3S

**Table 2:** Physical and chemical parameters assumed in the COMSOL computations performed in this work.

Desxription	Expression
Change density on Si_3_N_4_	− 0.02 [C / m^2^]
Change density on Au surface	0 [C / m^2^]
KCI concentration	0.3 M
Diffusion coefficient of K+	1.96 × 10^−9^ [m^2^. s^−1^]
Diffusion coefficient of CL−	2.03 × 10^−9^ [m^2^. S^−1^]
K + valence	1
Cl − Valence	−1
Laser power	20 mW
Wavelength	830 nm
Bias voltage	110 mV
AC voltage amplitude	+/− 110 mV
Frequencies range	lel − le 5 Hz
Solution viscosity	10^−3^ [Pa. s]
pH	7.4
KCL apace change density	9648 × 10^3^ [C/mol]
Vacuum permittivity	8.85 × 10^−12^ [F/m]
Temperature	300 [K]
Electrolyte rekative permitticity	80
Electrolyte electric conductivity	12.8 [S/m]

## Data Availability

Data will be made available on request.
